# Pregnancy Outcomes in Patients with Urosepsis and Uncomplicated Urinary Tract Infections—A Retrospective Study

**DOI:** 10.3390/medicina59122129

**Published:** 2023-12-07

**Authors:** Viorel-Dragos Radu, Petronela Vicoveanu, Alexandru Cărăuleanu, Ana-Maria Adam, Alina-Sinziana Melinte-Popescu, Gigi Adam, Pavel Onofrei, Demetra Socolov, Ingrid-Andrada Vasilache, AnaMaria Harabor, Marian Melinte-Popescu, Ioana Sadiye Scripcariu, Elena Mihalceanu, Mariana Stuparu-Cretu, Valeriu Harabor

**Affiliations:** 1 Urology Department, ‘Grigore T. Popa’ University of Medicine and Pharmacy, 700115 Iasi, Romaniaonofrei.pavel@gmail.com (P.O.); 2 Department of Mother and Child Care, ‘Grigore T. Popa’ University of Medicine and Pharmacy, 700115 Iasi, Romania; demetrasocolov@gmail.com (D.S.); isscripcariu@gmail.com (I.S.S.); emih2001@yahoo.com (E.M.); 3 Clinical and Surgical Department, Faculty of Medicine and Pharmacy, ‘Dunarea de Jos’ University, 800216 Galati, Romania; adam.anamaria89@gmail.com (A.-M.A.); haraboranamaria@yahoo.com (A.H.);; 4 Department of Mother and Newborn Care, Faculty of Medicine and Biological Sciences, ‘Ștefan cel Mare’ University, 720229 Suceava, Romania; alina.melinte@usm.ro; 5 Department of Pharmaceutical Sciences, Faculty of Medicine and Pharmacy, ‘Dunarea de Jos’ University, 800216 Galati, Romania; adam.gigi20@gmail.com (G.A.); marianascretu@yahoo.com (M.S.-C.); 6 Department of Internal Medicine, Faculty of Medicine and Biological Sciences, ‘Ștefan cel Mare’ University, 720229 Suceava, Romania

**Keywords:** urinary tract infections, urosepsis, pregnancy, preterm birth, premature rupture of membranes

## Abstract

*Background and Objectives*: Urinary tract infections (UTIs) are an important cause of perinatal and maternal morbidity and mortality. The aim of this study was to describe and compare the main pregnancy outcomes among pregnant patients with complicated and uncomplicated UTIs; *Materials and Methods*: This retrospective study included 183 pregnant patients who were evaluated for uncomplicated UTIs and urosepsis in the Urology Department of ‘C.I. Parhon’ University Hospital, and who were followed up at a tertiary maternity hospital—‘Cuza-voda’ from Romania between January 2014 and October 2023. The control group (183 patients) was randomly selected from the patient’s cohort who gave birth in the same time frame at the maternity hospital without urinary pathology. Clinical and paraclinical data were examined. Descriptive statistics and a conditional logistic regression model were used to analyze our data. *Results*: Our results indicated that patients with urosepsis had increased risk of premature rupture of membranes (aOR: 5.59, 95%CI: 2.02–15.40, *p* < 0.001) and preterm birth (aOR: 2.47, 95%CI: 1.15–5.33, *p* = 0.02). We could not demonstrate a statistically significant association between intrauterine growth restriction and pre-eclampsia with the studied urological pathologies. *Conclusions*: Careful UTI screening during pregnancy is needed for preventing maternal–fetal complications.

## 1. Introduction

Urinary tract infections (UTIs) are a common finding during pregnancy that could lead to important perinatal and maternal morbidity and mortality [1]. Due to heterogenous epidemiological reports, the current incidence of UTIs in pregnancy is estimated to fall in the 4–47% interval around the globe [2,3,4]. Several risk factors and physiologic changes in pregnancy play an important role in the development and spread of UTIs. The current literature has identified as predisposing factors for UTIs the following: ethnicity, advanced maternal age, multiparity, hydronephrosis in pregnancy, personal history of UTI, immunosuppressive conditions (e.g., diabetes mellitus), anemia, smoking, and low educational level or socioeconomic status [5,6].

The maternal complications associated with UTIs include chorioamnionitis, premature rupture of membranes, preterm labor, hypertensive disorders of pregnancy (such as pregnancy-induced hypertension and pre-eclampsia), and anemia [7,8,9].

Another important complication associated with complicated UTIs is represented by preterm birth. It can be also determined by multiple pregnancies, congenital abnormalities, chronic maternal conditions (diabetes, hypertension, autoimmune disorders, abdominal or uterine tumors, etc.), in vitro fertilization (IVF), extremes of ages or weight, maternal use of illicit drugs or alcohol, psychiatric comorbidities, or current pregnancy complications that require an iatrogenic preterm birth [10,11,12,13,14,15,16].

Neonatal complications that are associated with UTIs include sepsis and pneumonia (specifically, group B Streptococcus infection), intrauterine growth restriction, stillbirth, and a higher rate of neonatal intensive care unit (NICU) admissions [17,18,19].

In the presence of typical urinary symptoms, such as dysuria, frequency, and urgency, it is mandatory to perform a urine culture, regardless of the gestational age. Moreover, routine urinalysis during pregnancy can detect the presence of UTIs, even before they become symptomatic [20,21]. *Escherichia coli* (*E. coli*), *Proteus mirabilis*, *Klebsiella pneumoniae* (*K. pneumoniae*), group B *Streptococcus* (GBS), and *Staphylococcus saprophyticus* are among the most commonly identified pathogens in the pregnant patients’ urinary culture UTIs [22,23]. On the other hand, less common organisms, such as *Enterococci*, *Chlamydia trachomatis*, and *Ureaplasma urealyticum*, can also determine UTIs [24,25].

UTIs comprise a wide range of pathological entities that can be represented by asymptomatic bacteriuria, cystitis, pyelonephritis, and that can complicate with renal abscesses or urosepsis. Sepsis is a systemic host response to infection that causes acute organ failure and is primarily caused by the source of infection [26].

The maternal sepsis definitions underwent several revisions over time. In 1991, a set of criteria was developed, characterizing sepsis in terms of the inflammatory response and other host physiologic characteristics [27]. There were four categories, beginning with systemic inflammatory response syndrome (SIRS) and ending with septic shock.

Evidence-based recommendations on sepsis and septic shock were developed by the Surviving Sepsis Campaign (SSC) in 2002 to enhance the diagnosis accuracy of sepsis and minimize mortality [28]. Early treatment with intravenous antibiotics and appropriate fluid resuscitation was emphasized in these recommendations. Within the treatment plans, physicians were expected to use a method known as “early, goal-directed therapy” (EGDT) to reach predetermined hemodynamic targets [29]. SSC recommendations have been updated many times since their first publication between 2004 and 2016 [30].

Recommendations from a task team led to changes in sepsis nomenclature in 2016. Because of the complexity and potential mortality of sepsis, new criteria have been developed to distinguish it from simple infection. The Third International Consensus Definitions for Sepsis and Septic Shock, published in 2016, included the sepsis-related organ failure assessment (SOFA) and the quick sepsis-related organ failure assessment (qSOFA), both of which use SIRS as their guiding diagnostic principle [31]. Due to changes made to the definition in 2016, SIRS was no longer a diagnostic category [30]. Septic shock was redescribed as “life-threatening organ failure induced by a dysregulated host response to infection”, and the phrase “severe sepsis” was eliminated from usage, since it was unnecessary.

The World Health Organization (WHO) developed an evidence-based definition of maternal sepsis soon after the 2016 updates on sepsis [32]. Maternal sepsis was defined as “a life-threatening illness with organ failure originating from infection during pregnancy, delivery, postabortion, or the postpartum period”, which is consistent with the sepsis criteria used in nonpregnant individuals in 2016.

Urosepsis is a potential life-threatening condition, associated with important mortality and morbidity. It is estimated that between 9% and 31% of all sepsis cases are represented by urosepsis [33]. A prospective study in Ireland on 150,043 pregnant patients reported an incidence of maternal sepsis of 1.81 per 1000 pregnant women [34]. The same study indicated that the majority of maternal sepsis cases were antenatally diagnosed (46 out of 272 cases), and urosepsis was identified in 21 cases (45.6%), being the main responsible for maternal sepsis. Escherichia coli caused 55% of all antenatally diagnosed sepsis, followed by GBS infection (4%).

Management of maternal urosepsis includes early antibiotic initiation, fluid resuscitation, and administration of vasopressors [35]. A prompt recognition of maternal urosepsis is extremely important in order to prevent adverse pregnancy outcomes, such as preterm birth and perinatal mortality [36,37].

Given limited data regarding the pregnancy outcomes in patients with simple UTIs or complicated UTIs, specifically urosepsis, we have designed our research as a retrospective study which aimed to describe and compare the pregnancy outcomes in these groups of patients compared with controls.

## 2. Materials and Methods

Between January 2014 and October 2023, we performed retrospective observational research on pregnant women who had urosepsis and uncomplicated UTIs at the Urology Department of the ‘C.I. Parhon’ University Hospital in Iasi, Romania. All patients were followed up at a tertiary maternity hospital—‘Cuza-Voda’, Iasi, Romania. The control group comprised of women who gave birth at the same time as the other patients but had no urological disorders

This study was approved by Institutional Ethical Review Boards at both the ‘Cuza-Voda’ Maternity Hospital (no. 6778/24.08.2022) and the ‘C.I. Parhon’ University Hospital (no. 1808/04.03.2022). All individuals provided informed consent before to inclusion in the research. All procedures were performed in compliance with applicable regulations and standards.

We conducted a thorough data collection and analysis by evaluating the medical records of participants. Exclusion criteria were represented by pregnant patients with asymptomatic bacteriuria, who had multifetal gestations, ectopic pregnancies, first and second trimester abortions, intrauterine fetal death, fetuses with genetic or anatomic abnormalities, intrauterine infection, incomplete medical records, or who could not provide informed consent due to various motifs (age less than 18 years old, intellectual deficits, psychiatric comorbidities, etc.).

A total of 62 pregnant patients were diagnosed with urosepsis at ‘C.I. Parhon’ University Hospital, and were included in the first study group. The second study group comprised 121 pregnant patients with uncomplicated urinary tract infections, while the third group (controls) included 183 patients without urinary tract infection.

The following data were recorded: demographic data, the patient’s comorbidities, laboratory results (leucocyte number, CRP, procalcitonin, estimated glomerular filtration rate-eGFR, and urine culture result), associated medical treatment, type of complications, and pregnancy outcomes (type of birth, newborn’s gender, Apgar score, preterm birth, premature rupture of membranes, fetal growth restriction, preeclampsia, thrombotic complications, neonatal intensive care unit (NICU) admission, fetal death, and the presence of postpartum UTIs and vaginal infections).

The estimated glomerular filtration rate was calculated using the modification of diet in renal disease (MDRD) formula using age, sex, race, and serum creatinine concentration as parameters [38].

A 5 mL blood sample was retrieved from patients for PCT and CRP determination. Procalcitonin was measured in 1 mL of serum using the electrochemiluminescence immunoassay ECLIA (Elecsys BRAHMS PCT, Roche, Indianapolis, IN, USA), with a limit of detection of 0.02 ng/mL. A latex immunoturbidimetric assay (Hycount 5—CRP, Hycel Handelsgesellschaft m.b.H., Schwechat, Austria) was used to determine CRP values from a 0.5 mL of serum, using a detection limit of 0.15 mg/L.

Diagnostic criteria for acute pyelonephritis were flank discomfort, nausea or vomiting, a high fever (>38 °C), and/or soreness at the costovertebral angle, regardless of the presence or absence of cystitis symptoms [39]. A positive urine culture was defined in the presence of more than 105 colony-forming units (CFU)/mL. Urinary pathogens and their sensibility to antibiotics were determined by urine culture and antibiogram. According to the quick sepsis-related organ failure assessment (qSOFA), urosepsis was identified in individuals who met at least two of the following criteria: (1) a respiratory rate of 22 breaths/min or more; (2) altered consciousness (Glasgow coma scale score of less than 13); (3) systolic blood pressure of 100 mmHg or less [30]. We also calculated the sequential organ failure assessment (SOFA) score for those patients admitted in the intensive care unit [40].

The screening of infections in newborns was required in the case of maternal proven or suspected infections (urosepsis, UTIs, vaginal infections, pneumonia, etc.), preterm birth, premature rupture of membranes, prolonged rupture of membranes or prolonged labor, the presence of meconium in the amniotic fluid, or clinical suspicion of neonatal infection (fever, tachycardia, elevated inflammatory markers, etc.). This screening consisted in the analysis of biological samples from the external auditory canal, skin, blood (hemocultures), urine, or nasopharynx.

Statistical analysis was performed using STATA SE software (version 17, 2021, StataCorp LLC, College Station, TX, USA). The relationship between unfavorable pregnancy outcomes and forms of urinary tract infections was evaluated using a conditional logistic regression (CLR) model, and the adjusted odds ratios (aOR) with 95% confidence intervals (CI) were obtained for each variable of interest. A *p* value less than 0.05 was considered statistically significant.

## 3. Results

A total of 183 pregnant women with urological disorders who were admitted at ‘C.I. Parhon’ University Hospital during our study period were included in our study. A group of 183 patients, who gave birth at ‘Cuza Voda’ Hospital, without urological illnesses and interventions during pregnancy served as our control group. The demographic characteristics, comorbidities, and pregnancy outcomes of cases and controls are presented in Table 1.

Our data indicated that patients with urosepsis had significantly more immunosuppressive conditions (*p* < 0.001), ureterohydronephrosis (*p* < 0.001), pyelonephritis (*p* < 0.001), and nephrolithiasis (*p* = 0.04) compared to other groups. Moreover, the time interval between the onset of urinary symptoms and hospital admission for further investigations was higher for patients with urosepsis compared to patients with uncomplicated UTIs (5.00 ± 1.72 versus 2.78 ± 1.31, *p* < 0.001).

The gestational age at diagnosis was significantly higher for patients with uncomplicated UTIs compared patients with urosepsis (29.17 ± 5.44 versus 25.47 ± 6.29 weeks, *p* = 0.01). Regarding pregnancy outcomes, patients with urosepsis had significantly more premature rupture of membranes (*p* < 0.001) and preterm births (*p* < 0.001) compared to other groups. Moreover, patients with urosepsis gave birth to newborns who had significantly more neonatal intensive care unit (NICU) admissions rates (*p* < 0.001). No maternal or fetal death was recorded in our study. The rates of thrombotic complications were similar among groups.

The rates of postpartum UTIs (*p* < 0.001) and vaginal infections (*p* < 0.001) were significantly higher for patients with a history of urosepsis. The most frequent pathogens responsible for postpartum UTIs in this group were *Escherichia coli* (10 cases, 50%), *Klebsiella* spp. (3 cases, 15%), *Enterococcus* spp. (3 cases, 15%), followed by *Serratia* spp. (2 cases, 10%) and *Staphylococcus* spp. (2 cases, 10%). These UTIs were treated with amoxicillin–clavulanic acid 1 g b.i.d or cefuroxime 1.5 g b.i.d in the postpartum period for 10–14 days.

On the other hand, the most frequent pathogens that determined postpartum vaginal infections were *Candida albicans* (three cases, 21.4%), *Escherichia coli* (two cases, 14.2%), *Klebsiella* spp. (one case, 7.1%), and *Proteus mirabilis* (one case, 7.1%). The fungal vaginal infections were treated with vaginal suppositories of nystatin 100,00 UI or fluconazole 150 mg q.d. for 10 days. For the remaining vaginal infections, we administered amoxicillin–clavulanic acid 1 g b.i.d or cefuroxime 1.5 g b.i.d in the postpartum period for 10–14 days.

The paraclinical characteristics of the patients with urological disorders during admission to the urology department are presented in Table 2. The mean values and standard deviations (SDs) of pretreatment leukocytosis and serum CRP were significantly higher for patients diagnosed with urosepsis compared to the second group (leucocytes: 18,916.67 ± 3356.74/mm^3^ versus 14,691 ± 2636.38/mm^3^, *p* < 0.001; CRP: 131.38 ± 70.72 mg/L versus 81.7 ± 70.34 mg/L, *p* = 0.004). The same findings were determined in case of post-treatment values of leukocytosis and serum CRP (leucocytes: 15,672.78 ± 3582.62/mm^3^ versus 12,177.78 ± 2076.41/mm^3^, *p* < 0.001; CRP: 88.30 ± 56.34 mg/L versus 63.49 ± 44.02 mg/L, *p* = 0.041).

The main pathogens that determined uncomplicated urinary tract infection were *Escherichia coli* (n = 87; 71.9%) and *Streptococcus* spp. (n = 17; 14%), followed by *Klebsiella* spp. (n = 10; 8.2%) and *Staphylococcus* spp. (n = 6; 4.9%). On the other hand, the main pathogens that determined urosepsis were *Escherichia coli* (n = 38; 61.2%), *Klebsiella* spp. (n = 12; 19.3%), *Enterococcus* spp. (n = 3; 4.8%), *Serratia* spp. (n = 2; 3.2%), and *Staphylococcus* spp. (n = 2; 3.2%).

Uncomplicated urinary tract infections were treated with amoxicillin–clavulanic acid 1 g b.i.d or cefuroxime 1.5 g b.i.d in the postpartum period for 10–14 days. Urosepsis cases were evaluated in the intensive care unit, and received Ceftriaxone 2 g b.i.d, piperacillin/tazobactam 4.5 g t.i.d, or meropenem 1 g t.i.d (for multiresistant bacteria), along with supportive treatment.

The bacterial spectrum of neonatal infections in the first group was represented by *Escherichia coli* (n = 5; 71.4%), *Klebsiella* spp. (n = 1; 14.3%), and *Staphylococcus* spp. (n = 1; 14.3%), while in the second group, *Escherichia coli* (n = 11; 73.3%), *Streptococcus* spp. (n = 2; 13.3%), and *Klebsiella* spp. (n = 2; 13.3%) determined neonatal infections.

The results of the CLR model with adjusted odds ratios are displayed in Table 3. The presence of a urosepsis diagnosis during pregnancy was associated with increased odds of the premature rupture of membranes (aOR: 5.59, 95%CI: 2.02–15.40, *p* < 0.001) and preterm birth (aOR: 2.47, 95%CI: 1.15–5.33, *p* = 0.02). On the other hand, none of the urological disorders were associated with adverse pregnancy outcomes, such as intrauterine growth restriction (*p* = 0.66), pre-eclampsia (*p* = 0.22), NICU admission (*p* = 0.06), or ARDS (*p* = 0.42).

## 4. Discussion

Urinary sepsis during pregnancy is a poorly explored topic in the literature, and in this retrospective study, we presented our clinical experience over almost 8 years regarding the pregnancy outcomes in patients who developed uncomplicated UTIs or urosepsis compared with controls. Our results indicated that patients with urosepsis had an increased likelihood of premature rupture of membranes (aOR: 5.59, 95%CI: 2.02–15.40, *p* < 0.001) and preterm birth (aOR: 2.47, 95%CI: 1.15–5.33, *p* = 0.02). The role of urinary infections in the determinism of preterm birth is well established, and immunosuppressive conditions favor the development of severe forms of infection, as we determined in this study [41,42,43,44,45].

Intrauterine growth restriction and pre-eclampsia are two pathologic entities that derive from the ischemic placental disorder. Both conditions have been associated in the literature with the occurrence of urinary tract infections [46]. A population-based study by Mazor-Dray et al., on a cohort of 199,099 deliveries, demonstrated that patients with UTIs had significantly higher rates of intrauterine growth restriction, pre-eclampsia, caesarean deliveries, and preterm deliveries [18]. Although our results confirmed the association between urosepsis and preterm deliveries, we could not demonstrate a statistically significant association between the other pregnancy outcomes, such as IUGR and pre-eclampsia, mainly due to a small cohort of patients. It is important to note that the current data in the literature are scarce regarding the description of adverse pregnancy outcomes in the urosepsis context.

Another finding of this study showed that patients with urosepsis gave birth to newborns who had significantly more NICU admission rates. This observation might be validated by the fact that preterm newborns often experience increased morbidity and need specialized care in NICUs [47,48]. Moreover, it was demonstrated that maternal UTI during pregnancy increases the risk of congenital malformations [49,50], but this aspect was not observed in our cohort of patients. Also, no neonatal deaths were recorded in the study groups.

Adverse pregnancy outcomes were more prevalent in both urosepsis and uncomplicated urinary tract infections cases compared to controls, but our results indicated that a previous diagnosis of urosepsis in pregnancy significantly increased the odds of the premature rupture of membranes and preterm birth.

Thus, careful monitoring of pregnant patients, especially in the presence of risk factors, such as nephrolithiasis, ureterohydronephrosis, and double-J ureteric stenting, is extremely important for the early detection of UTIs and for preventing their progression to infectious complications

Both patients who developed uncomplicated UTIs and urosepsis presented risk factors for such conditions, but they were more prevalent in the urosepsis group. For example, in our study, we found that patients with urosepsis had significantly more diagnoses of ureterohydronephrosis, pyelonephritis, nephrolithiasis, and double-J ureteric stenting. Moreover, the time interval between the onset of urinary symptoms and hospital admission for further investigations was higher for patients with urosepsis compared to patients with uncomplicated UTIs. All these are known risk factors for developing UTIs in pregnancy [6,51], and we hypothesize that the longer timeframe until admission to the urology department in the case of patients with urosepsis constitutes an important aggravating factor.

*Escherichia coli, Klebsiella* spp., and *Enterococcus* spp. were the main determinants of UTIs in our cohort of patients. This bacterial spectrum corresponds to the pathogenic microorganisms associated with UTI in pregnancy [43,52,53]. Postpartum UTIs were more frequently encountered in the urosepsis group, and the bacterial spectrum responsible for postpartum UTI was similar to that described by the urine culture taken during pregnancy, with *Escherichia coli, Klebsiella* spp., and *Enterococcus* spp. being the most prevalent.

Procalcitonin is a serum prohormone that increases in the presence of endotoxins and proinflammatory mediators in the context of tissue damage, systemic inflammation, and especially severe bacterial infection [54]. It is a relatively new biomarker that outperforms the traditional biomarkers, such as C-reactive protein and leukocyte count, when used for the diagnosis of bacterial infection and for the monitoring of antibiotic therapy [55,56].

Although CRP is not a very specific test for sepsis, it does have a very high negative predictive value [57]. Moreover, it has a longer latent period when compared with PCT (6 versus 2 h) [58,59]. The PCT serum levels rapidly increase during the first hours of severe bacterial infections and can support the decision for early antibiotic administration in severe bacterial infections [60].

Due to its elevated levels during pregnancy, leucocyte count has limited potential as a biomarker of infection and inflammation [61]. On the other hand, it demonstrated a significant rise in PCT serum levels in Gram-negative infections compared to Gram-positive [62]. In cases of severe obstetric infections, E. coli is the most often detected pathogen in blood cultures [34]. A PCT value between 2 and 10 ng/mL indicates that a systemic infection is possible, and in our study, the mean PCT value and standard deviation for patients in the urosepsis group were 5.22 ± 1.35 ng/mL. Also, the CRP and leucocyte values were significantly higher for the urosepsis group compared to the second group.

Acute kidney injury (AKI) can be a result of a septic state. A prospective observational study by Gopalakrishnan et al., on 130 patients, reported an incidence of acute kidney injury in pregnancy of 7.8%, and it was determined in the majority of cases (39%) by maternal sepsis [63]. However, since the RIFLE (risk, injury, failure, loss, and end-stage) criteria, the KDIGO (Kidney Disease Improving Outcomes) recommendations, and the AKI Network (AKIN) criteria have not been validated in the pregnant population, the prevalence of pregnancy-related AKI is difficult to ascertain [64,65].

No maternal and fetal deaths were recorded in our cohort of patients, and this finding is in line with previous reports [34]. However, obstetrical sepsis remains an important cause of mortality, with a rate between 8% and 14% for patients with septic shock [35,40,66]. Shields et al. outlined the idea that clinicians should maintain a high index of suspicion for maternal sepsis, especially in the context of nonspecific symptoms [67]. This perspective would allow them to promptly apply sepsis scoring systems and to initialize early antibiotic treatment.

The high cesarean delivery rates could be partially explained by the iatrogenic delivery of the fetus in the case of altered fetal status (acute fetal distress, chorioamnionitis, premature rupture of membranes, etc.) and/or maternal decompensation, despite prompt antibiotherapy and double-J ureteric stenting. Furthermore, previous cesarean delivery in the personal history of our patients, along with the patients’ desire to avoid vaginal delivery, were also factors that influenced the high cesarean delivery rates.

The main limitation of our study is the small cohort of patients. On the other hand, this study retrospectively identified urosepsis cases that were treated in a regional center from Romania in an 8-year time-frame, and could serve as a basis for other observational studies on larger cohorts of patients.

## 5. Conclusions

Early identification of risk factors that promote urinary tract complications, especially during pregnancy, could determine a shift in the monitoring and therapeutic approaches towards a more active stance.

Moreover, clinicians could include these risk factors in the counseling sessions of pregnant patients and inform them about the possible adverse outcomes.

## Figures and Tables

**Table 1:** Demographic characteristics, comorbidities, and pregnancy outcomes of the evaluated groups.

Variable		Group 1 (n = 62 Patients with Urosepsis)	Group 2 (n = 121 Patients with Uncomplicated UTI)	Group 3 (n = 183 Patients as Controls)	*p* Value
Demographic characteristics	Age, years (mean ± SD)	29.6 ± 6.19	26.12 ± 5.74	27.8 ± 8.11	0.52
	Medium (n/%)	Rural = 32 (51.6%) Urban = 30 (48.3%)	Rural = 64 (52.8%) Urban = 57 (47.1%)	Rural = 107 (58.4%) Urban = 76 (41.5%)	0.86
	Parity (mean ± SD)	2.06 ± 2.08	1.56 ± 0.5	1.36 ± 0.48	0.06
Comorbidities	Previous cesarean section (n/%)	Yes—10 (16.1%)	Yes—30 (24.7%)	Yes—46 (25.1%)	0.32
	Immunosuppression (n/%)	Yes—20 (32.2%)	Yes—17 (14%)	Yes—10 (5.4%)	<0.001
	UHN (n/%)	Yes—53 (85.4%)	Yes—33 (27.2%)	Yes—0 (0%)	<0.001
	UHN grade (n/%) *	I—12 (22.7%) II—34 (64.1%) III—7 (13.2%)	I—6 (18.8%) II—20 (60.6%) III—3 (21.2%)	-	<0.001
	UHN location (n/%) *	Left—9 (16.9%) Right—31 (58.4%) Bilateral-13 (24.5%)	Left—7 (21.2%) Right—23 (69.6%) Bilateral-3 (9%)	-	<0.001
	Nephrolithiasis (n/%)	Yes—9 (14.5%)	Yes—7 (5.7%)	-	0.04
	Pyelonephritis (n/%)	Yes—35 (56.4%)	Yes—0 (0%)	-	<0.001
	Double-J ureteric stenting	Yes—14 (22.5%)	Yes—6 (4.9%)	-	<0.001
	Gestational age at diagnosis, weeks (mean ± SD)	25.47 ± 6.29	29.17 ± 5.44	-	0.010
	Time interval from symptoms to admission, days (mean ± SD)	5.00 ± 1.72	2.78 ± 1.31	-	<0.001
	Time interval from admission to delivery, days (mean ± SD)	7.16 ± 3.37	27.78 ± 14.23		<0.001
Pregnancy outcomes	Type of birth (n/%)	Cesarean—41 (66.1%) Vaginal—21 (33.8%)	Cesarean—83 (68.5%) Vaginal—38 (31.4%)	Cesarean—116 (63.3%) Vaginal—67 (36.6%)	0.88
	Birthweight, g (mean ± SD)	2938.8 ± 693.67	3037.2 ± 509.3	3236.11 ± 398.5	0.068
	Apgar score at 1 min (mean ± SD)	7.69 ± 1.68	8.14 ± 1.98	8.47 ± 0.73	0.11
	Gestational age at birth (mean ± SD)	36.2 ± 2.93	37.5 ± 2.04	38.5 ± 1.13	0.03
	Preterm birth (n/%)	Yes—17 (27.4%)	Yes—16 (13.2%)	Yes—10 (5.4%)	<0.001
	Premature rupture of membranes (n/%)	Yes—14 (22.5%)	Yes—6 (4.9%)	Yes—8 (4.3%)	<0.001
	Fetal growth restriction (n/%)	Yes—8 (12.9%)	Yes—13 (10.7%)	Yes—15 (8.1%)	0.51
	Pre-eclampsia (n/%)	Yes—6 (9.6%)	Yes—6 (4.9%)	Yes—1 (0.5%)	0.001
	Thrombotic complications (n/%)	Yes—3 (4.8%)	Yes—3 (2.4%)	Yes—1 (0.5%)	0.08
	NICU admission (n/%)	Yes—17 (27.4%)	Yes—19 (15.7%)	Yes—8 (4.3%)	<0.001
	ARDS (n/%)	Yes—8 (12.9%)	Yes—11 (9.09%)	Yes—6 (3.2%)	0.01
	Neonatal infections (n/%)	Yes—7 (11.2%)	Yes—15 (12.3%)	Yes—15 (8.1%)	0.46
	Postpartum UTI (n/%)	Yes = 20 (32.2%)	Yes—7 (5.7%)	Yes—3 (1.6%)	<0.001
	Postpartum vaginal infections (n/%)	Yes = 14 (22.5%)	Yes—4 (3.3%)	Yes—16 (8.7%)	<0.001

UTI—urinary tract infection; SD—standard deviation; UHN—ureterohydronephrosis; NICU—neonatal intensive care unit; ARDS—acute respiratory distress syndrome; * from patients who developed ureterohydronephrosis.

**Table 2:** Paraclinical characteristics of the patients with urological disorders during admission to the urology department.

Variable	Group 1 (n = 62 Patients with Urosepsis)	Group 2 (n = 121 Patients with Uncomplicated UTI)	*p* Value
Pretreatment leukocyte number/mm^3^ (mean ± SD)	18,916.67 ± 3356.74	14,691 ± 2636.38	<0.001
Post-treatment leukocyte number/mm^3^ (mean ± SD)	15,672.78 ± 3582.62	12,177.78 ± 2076.41	<0.001
Pretreatment CRP value, mg/dL (mean ± SD)	131.38 ± 70.72	81.7 ± 70.34	0.004
Post-treatment CRP value, mg/dL (mean ± SD)	88.30 ± 56.34	63.49 ± 44.02	0.041
Procalcitonin, ng/mL (mean ± SD)	5.22 ± 1.35	-	-
qSOFA score (mean ± SD)	2.25 ± 0.64	-	-
SOFA score (mean ± SD)	9.16 ± 0.32		

UTI—urinary tract infection; SD—standard deviation; CRP—C-reactive protein; eGFR—estimated glomerular filtration rate; (q)SOFA—(quick) sepsis-related organ failure assessment.

**Table 3:** Results from conditional logistic model including patients with urological disorders and adverse pregnancy outcomes.

Adverse Pregnancy Outcome	aOR	95% Confidence Interval Lower Limit	95% Confidence Interval Upper Limit	*p* Value
Preterm birth	2.47	1.15	5.33	0.02
PPROM	5.59	2.02	15.40	<0.001
IUGR	1.23	0.48	3.14	0.66
Pre-eclampsia	2.07	0.63	6.65	0.22
NICU	2.02	0.96	4.26	0.06
ARDS	1.48	0.56	3.89	0.42

aOR—adjusted odds ratio; UTI—urinary tract infection; PPROM—premature rupture of membranes; IUGR—intrauterine growth restriction; NICU—neonatal intensive care unit admission; ARDS—acute respiratory distress syndrome.

## Data Availability

The data presented in this study are available on request from the corresponding authors. The data are not publicly available due to local policies.
